# Determinants of Severe Acute Malnutrition Among Children Aged 6–23 Months Attending Public Hospitals in the Benadir Region, Somalia: An Unmatched Case–Control Study

**DOI:** 10.1002/hsr2.72897

**Published:** 2026-07-25

**Authors:** Muna Hassan Warsame, Sade Bashir Hassan, Jamal Hassan Mohamoud, Ahmed Mahad Sheikh Mohamed, Mohamed Hussein Adam, Bashiru Garba, Mohamed Adam Mahamud

**Affiliations:** ^1^ Department of Public Health, Faculty of Medicine and Health Sciences SIMAD University Mogadishu Somalia; ^2^ SIMAD Institute for Global Health (SIGHt) SIMAD University Mogadishu Somalia; ^3^ Department of Microbiology and Laboratory Sciences, Faculty of Medicine and Health Sciences SIMAD University Mogadishu Somalia

**Keywords:** complementary feeding, risk factors Somalia, severe acute malnutrition

## Abstract

**Background and Aims:**

Severe acute malnutrition (SAM) remains a leading cause of morbidity and mortality among children under 2 years in Somalia. This study aimed to identify determinants of SAM using a hospital‐based unmatched case–control design.

**Methods:**

An unmatched case–control study was conducted from February to May 2025 at two public hospitals in Mogadishu. A total of 255 children (85 cases and 170 controls) aged 6–23 months were included. Statistical comparisons between groups were performed using chi‐square tests, and multivariable logistic regression identified independent predictors. Adjusted odds ratios (AORs) with 95% confidence intervals (CIs) were reported.

**Results:**

Significant differences between cases and controls were observed across several variables. Large family size (AOR = 1.92; 95% CI: 1.24–4.08), short birth interval < 2 years (AOR = 4.11; 95% CI: 2.98–17.19), unprotected water source (AOR = 2.24; 95% CI: 1.37–4.11), and use of traditional cooking fuel (AOR = 6.48; 95% CI: 2.18–19.24) were independent predictors. Early (< 6 months) and delayed (> 6 months) complementary feeding were analyzed separately, both showing increased risk, with delayed initiation demonstrating a stronger association. Presence of comorbid illness (AOR = 1.96; 95% CI: 1.07–3.84) was also associated with SAM.

**Conclusion:**

SAM is influenced by multifactorial determinants including household, environmental, and feeding practices. Targeted interventions are essential to reduce malnutrition burden.

## 1 Background

Severe acute malnutrition (SAM), referred to as severe wasting, is defined by one or more of the following criteria: a weight‐for‐length (WFL) z‐score less than –3 standard deviations (SD) from the median of the World Health Organization (WHO) growth standards; a mid‐upper arm circumference (MUAC) of less than 11.5 cm; or the presence of bilateral pitting edema [1]. Globally, approximately 50 million children suffer from acute malnutrition, with 17 million classified as severely wasted. Undernutrition is estimated to contribute to about 45% of all child deaths each year [2]. In Africa, approximately 14.1 million cases of child wasting have been reported, with about one‐third—equivalent to 4.3 million—classified as severe wasting. In Eastern Africa, 6.6% of children under the age of five have been identified as wasted due to malnutrition [3]. In 2025, an estimated 1.7 million children aged 6–59 months in Somalia are expected to suffer from acute malnutrition, including 466,000 with SAM. Approximately 64% of these cases are concentrated in southern regions. Although Global Acute Malnutrition (GAM) has declined by 1% compared to the previous year, SAM has increased by 9%, indicating a worsening in the severity of malnutrition [4].

Severe acute malnutrition (SAM) is the most severe and noticeable form of acute undernutrition. It typically arises from acute food insecurity, poor dietary quality, recent illness, recurrent infections, improper child care, or inadequate feeding practices—or a combination of these factors. As a result, affected children experience rapid weight loss and fail to gain sufficient weight in proportion to their height [5]. Although SAM can affect children of all age groups, but its impact is most severe among children aged 6 to 23 months. This age range is critical for optimal growth and development, and nutritional deficiencies during this period can lead to mental and physical impairments that are often difficult to reverse [6].

Moreover, many more factors like acute infections like diarrhea and malaria [7, 8], inappropriate initiation time of complementary feeding [9], water sources [10], age of the child [11], maternal age and nutrition counseling during pregnancy [11].

Since the collapse of its central government in 1991, Somalia has endured prolonged conflict and political instability, making it one of the most insecure and impoverished countries in the world. It currently ranks as the sixth poorest globally and records some of the highest rates of child and maternal mortality [12]. Somalia has adopted a National Nutrition Strategy (2020–2025), demonstrating a coordinated effort to address the country's high burden of malnutrition [13]. The strategy outlines evidence‐based interventions aimed at improving maternal and child nutrition and strengthening health outcomes. Its implementation is particularly critical given Somalia's longstanding challenges with food insecurity and undernutrition. However, the delivery of these interventions is largely dependent on humanitarian support. Recent suspension of operations by key partners like USAID has disrupted services and threatens nutrition program continuity.

There are significant gaps in understanding the association between SAM and factors such as complementary feeding practices, maternal characteristics, and environmental conditions. To address these gaps, the present study aimed to assess the determinants of SAM among children aged 6–23 months in the Benadir region, Somalia. Understanding the determinants of SAM in children aged 6–23 months is vital for designing targeted interventions, informing policy, and improving nutrition programs, particularly for this vulnerable age group where timely action is critical to reducing child morbidity and mortality.

## 2 Methods and Materials

### 2.1 Study Setting and Period

This study was conducted at SOS Mother and Child Hospital and Benadir Hospital, two major public healthcare institutions located in Mogadishu, the capital of Somalia, within the Benadir Region. These hospitals function as key referral centers and provide a broad range of maternal and pediatric healthcare services, including the diagnosis and treatment of SAM. Both facilities are among the largest and most accessible in the region, serving patients from both urban neighborhoods and rural areas. Their pediatric wards are specifically equipped and staffed to manage cases of SAM, making them suitable and reliable settings for conducting this study. The study was carried out from February 25 to May 20, 2025.

### 2.2 Study Design and Study Population

This study employed hospital‐based unmatched case‐control design. It was conducted among children aged 6–23 months who were admitted to the pediatric wards of SOS and Benadir hospitals. The study population included both children diagnosed with SAM (cases) and those without SAM (controls).

### 2.3 Cases

Cases were defined as children aged 6–23 months who met the World Health Organization's Integrated Management of Neonatal and Childhood Illness (IMNCI) criteria for severe acute malnutrition [14]. These children were identified by the presence of at least one of the following indicators: a mid‐upper arm circumference (MUAC) of less than 11.5 cm, a weight‐for‐length z‐score (WLZ) of less than –3 standard deviations (SD), or the presence of bilateral pitting edema. Diagnosis was confirmed through clinical assessment by pediatricians, and all cases were admitted to the pediatric units of the two selected public hospitals.

### 2.4 Controls

Controls were children aged 6–23 months who were admitted to the same hospitals during the study period but did not meet the criteria for SAM. These children had a MUAC greater than 12.5 cm, a WLZ of –2 SD or above, and no signs of bilateral pitting edema. They were admitted for other health conditions unrelated to malnutrition, and their nutritional status was assessed through history taking and anthropometric measurements.

## 3 Inclusion and Exclusion Criteria

The study included all children admitted to the inpatient (IPW) and outpatient pediatric wards (POD), as well as those visiting under‐five and well‐baby clinics during the study period. Children who were unconscious or had physical deformities or burns on their hands that made it difficult to take anthropometric measurements were excluded from both the case and control groups.

### 3.1 Study Variables

Severe acute malnutrition was the dependent variable, while the independent variables included a range of factors. Socio‐demographic factors covered maternal age, marital status, occupation, parental education, maternal employment status, monthly income, and place of residence. Child‐related factors included the child's age, sex, vaccination status, exclusive breastfeeding practices, and the age at which complementary feeding was introduced and comorbidities were defined as the presence of acute illnesses such as diarrheal, respiratory infections, or febrile conditions at the time of admission. Maternal factors involved the mother's age at first birth, antenatal care follow‐up, and place of delivery. Environmental factors included the source of drinking water, type of cooking fuel, method and location of waste disposal, availability of a toilet, and the type of housing.

### 3.2 Sample Size Calculations and Sample Technique

The sample size for this study was calculated using Epi Info version 7 software, based on the two‐population proportion formula. The calculation was performed with the following assumptions: a 95% confidence level, 80% power, and a case‐to‐control ratio of 1:2. The estimate was guided by findings from a previous study conducted in Nekemte, Oromia Region, which reported that 42.9% of children with wasting came from large families, with an associated odds ratio of 2.42 [15].

After accounting for a 10% non‐response rate, the final sample size was determined to be 255 participants, including 85 cases and 170 controls.

## 4 Sample Technique and Procedure

The sample size was proportionally allocated between SOS Mother and Child Hospital and Benadir Hospital, based on the average monthly number of cases involving children aged 6 to 59 months. At SOS Hospital, 41 cases and 90 controls were selected from a total of 94 cases and 190 controls using a systematic random sampling method, with every second child selected from the registration book, which served as the sampling frame. At Benadir Hospital, 44 cases and 80 controls were selected from 79 cases and 181 controls using the same sampling approach. In instances where a mother or caregiver declined to participate, the next eligible individual was approached those who met the inclusion criteria and consented to participate were enrolled in the study.

## 5 Data Quality Control

Data collectors received 4 days of training, focusing on proper questionnaire completion and accurate MUAC measurement to minimize observer errors. A pre‐test was conducted on 5% of the sample at Benadir Hospital, and adjustments were made accordingly. Data collectors worked in pairs under the supervision of the principal investigator. Each questionnaire was checked for completeness and consistency before data entry. MUAC measurements were taken using standard procedures by trained personnel.

## 6 Data Collection Instrument and Procedure

Data were obtained from all eligible participants using a structured questionnaire adapted from previously published sources [16, 17, 18, 19]. The tool encompassed a wide range of areas, including socioeconomic and demographic characteristics, childcare practices, maternal factors, and environmental health conditions. Mid‐upper arm circumference (MUAC) was measured using a portable MUAC tape to assess the nutritional status of children and to differentiate cases from controls. Anthropometric assessment was primarily based on MUAC measurements due to feasibility constraints in clinical settings; therefore, other indicators such as weight‐for‐length z‐scores (WLZ) were not included in the analysis. Complementary feeding timing was categorized into 2 groups: timely initiation (at 6 months), early initiation (< 6 months) or delayed initiation (> 6 months). To ensure linguistic accuracy and cultural appropriateness, the questionnaire was translated into Somali and then back‐translated into English by qualified language experts.

## 7 Data Analysis Procedures

All statistical analyses were conducted using SPSS version 29.0 (IBM Corp., Armonk, NY, USA). Descriptive statistics were reported as frequencies with corresponding numerators and denominators (n/N, %). Continuous variables were summarized using means ± standard deviations (SD) or medians with interquartile ranges (IQR), depending on distribution. Comparisons between cases and controls were performed using chi‐square tests or Fisher's exact tests where appropriate. Variables with *p* < 0.25 in bivariate analysis were included in multivariable logistic regression. A two‐sided *p*‐value < 0.05 was considered statistically significant. Effect sizes were presented using odds ratios (ORs) and adjusted odds ratios (AORs) with 95% confidence intervals (CIs). Model fit was assessed using the Hosmer–Lemeshow goodness‐of‐fit test.

## 8 Ethical Consideration

The study adhered to the ethical principles outlined in the Declaration of Helsinki. Ethical approval was obtained from the Institutional Review Board (IRB) of the Faculty of Medicine and Health Sciences, SIMAD University (Reference No. 2024/SU‐IRB/FMHS/P0032). Prior to participation, informed consent was obtained from all respondents after a clear explanation of the study's purpose and procedures. Participants were also assured that all information collected would remain strictly confidential and used solely for research purposes.

## 9 Results

### 9.1 Sociodemographic Characteristics among Mothers

A total of 255 mother–child pairs participated in the study, including 85 cases and 170 controls. Most mothers were aged 15–31 years, accounting for 69/85 (81.2%) of the cases and 148/170 (87.1%) of the controls, whereas only a small proportion were 16/85 (18.8%) and 22/170 (12.9%), respectively. Similarly, the majority were currently married, as reported by 68/85 (80.0%) of the cases and 144/170 (84.7%) of the controls. In terms of education, a higher proportion of mothers of the cases had no formal schooling, 65/85 (76.5%), than those of the controls, 110/170 (64.7%), while formal education was more frequent among the controls, 60/170 (35.3%), compared to 20/85 (23.5%) among cases. Regarding occupational status, unemployment was more common among cases, 65/85 (76.5%), than among controls, 118/170 (69.4%), whereas employment was slightly higher among controls, 52/170 (30.6%), compared to 20/85 (23.5%) among cases. In addition, households with five or more members were more common among cases (67/85, 78.8%) than among controls (91/170, 53.5%). Conversely, households with fewer than five members were more common among controls (79/170, 46.5%) than among cases (18/85, 21.2%). Moreover, low monthly income (< 50 $) was reported by 77/85 (90.6%) of cases and 160/170 (94.1%) of controls. Finally, urban residence was predominant in both groups, accounting for 80/85 (94.1%) of cases and 157/170 (92.4%) of controls (Table 1).

**Table 1:** Sociodemographic characteristics among mothers.

Parameters	Cases	Controls	*p* value
	*N* (%)	*N* (%)	
Maternal age (mean ± SD): 25.6 ± 6.5 years			
15–31 years	69 (81.2)	148 (87.1)	0.213
32–49 years	16 (18.8)	22 (12.9)	
Current marital status			
Currently married	68 (80.0)	144 (84.7)	0.895
Currently unmarried	17 (20.0)	26 (15.3)	
Maternal educational level			
No formal	65 (76.5)	110 (64.7)	0.056
Formal	20 (23.5)	60 (35.3)	
Maternal occupational status			
Employed	20 (23.5)	52 (30.6)	0.237
Unemployed	65 (76.5)	118 (69.4)	
Family size			
≥ 5	67 (78.8)	91 (53.5)	< 0.001
< 5	18 (21.2)	79 (46.5)	
Family income level ($)			
< 50	77 (90.6)	160 (94.1)	0.299
≥ 50	8 (9.4)	10 (5.9)	
Place of residence			
Urban	80 (84.1)	157 (92.4)	0.604
Rural	5 (5.9)	13 (7.6)	

## 10 Maternal Characteristics of Study Participant

The majority of mothers in both groups had other children, with 73/85 (85.9%) of cases and 138/170 (81.2%) of controls reporting additional children. Similarly, most had attended antenatal care (ANC) during pregnancy, with 74/85 (87.1%) of cases and 139/170 (81.8%) of controls. In terms of place of delivery, a higher proportion of cases delivered at home, 51/85 (60.0%), than controls, 58/170 (34.1%), whereas institutional deliveries were more common among controls, 112/170 (65.9%), compared to 34/85 (40.0%) among cases. Regarding nutrition counseling during ANC, 69/85 (81.2%) of mothers of cases and 132/170 (77.6%) of controls received guidance during ANC. In contrast, fewer mothers reported receiving postnatal nutrition counseling, particularly among cases, 43/85 (50.6%), than among controls, 102/170 (60.0%). Additionally, a shorter birth interval of less than 2 years was more common among cases, 82/85 (96.5%), than controls, 150/170 (88.2%). Longer intervals (≥ 2 years) were reported by only 3/85 (3.5%) of cases and 20/170 (11.8%) of controls (Table 2).

**Table 2:** Maternal characteristics of study participant.

Parameters	Cases	Controls	*p* value
	*N* (%)	*N* (%)	
Having other children			
Yes	73 (85.9)	138 (81.2)	0.348
No	12 (14.1)	32 (18.8)	
ANC follow up			
Yes	74 (87.1)	139 (81.8)	0.282
No	11 (12.9)	31 (18.2)	
Place of birth			
Home	51 (60.0)	58 (34.1)	0.008
Health institution	34 (40.0)	112 (65.9)	
Nutritional counselling during ANC			
Yes	69 (81.2)	132 (77.6)	0.515
No	16 (18.8)	38 (22.4)	
Nutritional counselling during PNC			
Yes	43 (50.6)	102 (60.0)	0.152
No	42 (49.4)	68 (40.0)	
Birth interval (in years)			
< 2 years	82 (96.5)	150 (88.2)	0.03
≥ 2 years	3 (3.5)	20 (11.8)	

## 11 Child Related Characteristics

In terms of child characteristics, female children represented a greater proportion among cases, 49/85 (57.6%), than among controls, 77/170 (45.3%), whereas male children were more common in the control group, 93/170 (54.7%), compared to 36/85 (42.4%) among cases. A history of malnutrition was reported in 41/85 (48.2%) of the cases and 65/170 (38.2%) of the controls. Regarding immunization status, only 18/85 (21.2%) of cases and 23/170 (13.5%) of controls were fully vaccinated. Partial vaccination was more frequent among controls, 133/170 (78.2%), than among cases, 53/85 (62.3%), whereas the proportion of unvaccinated children was higher among cases, 14/85 (16.5%), than among controls, 14/170 (8.2%). Exclusive breastfeeding was less common among cases, 51/85 (60.0%), than among controls, 135/170 (79.4%). Regarding the timing of complementary feeding, a greater proportion of controls introduced complementary foods after 6 months, 96/170 (56.5%), than the cases, 35/85 (41.2%). Conversely, timely introduction at 6 months was more frequent among cases, 50/85 (58.8%), than among controls, 18/170 (10.6%). Regarding comorbidities, 52/85 (61.2%) of cases had at least one comorbid condition, whereas 76/170 (44.7%) of controls reported the same. Finally, vitamin A supplementation coverage was high in both groups, although it was slightly higher among the controls, 153/170 (90.0%), than in the cases, 69/85 (81.2%) (Table 3).

**Table 3:** Child related characteristics among children aged 12–23 months.

Parameters	Cases	Controls	*p* value
	*N* (%)	*N* (%)	
Sex of child			
Male	36 (42.4)	93 (54.7)	0.062
Female	49 (57.6)	77 (45.3)	
History of malnutrition			
Yes	41 (48.2)	65 (38.2)	0.126
No	44 (51.8)	105 (61.8)	
Immunization status			
Fully vaccinated	18 (21.2)	23 (13.5)	0.712
Age appropriate	53 (62.3)	133 (78.2)	
Not vaccinated at all	14 (16.5)	14 (8.2)	
Exclusive breast feeding			
Yes	51 (60.0)	135 (79.4)	<0.001
No	34 (40.0)	35 (20.6)	
Initiation of complementary feeding			
At six months	50 (58.8)	152 (89.4)	<0.001
Before or after six months	35 (41.2)	18 (10.6)	
Comorbidities status			
Yes	52 (61.2)	76 (44.7)	0.013
No	33 (38.3)	94 (55.3)	

## 12 Environmental Characteristics

In this study, most households used safe drinking water sources. More control households, 163/170 (95.9%), used protected water than case households, 75/85 (88.2%). Unprotected water was more common in case households, 10/85 (11.8%), than in control households, 7/170 (4.1%). Most case households, 80/85 (94.1%), used traditional cooking fuels like wood or charcoal, while fewer control households, 90/170 (52.9%), did. Modern fuels like electricity or gas were used by 80/170 (47.1%) of control households but only 5/85 (5.9%) of case households. Waste disposal methods were different between the groups. Proper disposal was practiced by 25/85 (29.4%) of case households and 108/170 (63.5%) of control households. Improper disposal was more common in case households, 60/85 (70.6%), than in control households, 62/170 (36.5%). Almost all households had access to toilets, with 84/85 (98.8%) of cases and 168/170 (98.8%) of controls reporting availability. For waste disposal, landfills were used more by control households, 106/170 (62.4%), than by case households, 24/85 (28.2%). Burying, 32/85 (37.6%), and burning, 24/85 (28.2%), were more common in case households than in control households, 30/170 (17.6%) and 28/170 (16.5%), respectively (Table 4).

**Table 4:** Environmental characteristics among study participants.

Parameters	Cases	Controls	*p* value
	*N* (%)	*N* (%)	
Source of drinking water			
Protected	75 (88.2)	163 (95.9)	0.021
Unprotected	10 (11.8)	7 (4.1)	
Types of coking fuel			
Modern	5 (5.9)	80 (47.1)	<0.001
Traditional	80 (94.1)	90 (52.9)	
Methods of waste disposal			
Proper	25 (29.4)	108 (63.5)	<0.001
Improper	60 (70.6)	62 (36.5)	
Having toilet			
Yes	84 (98.8)	168 (98.8)	0.2341
No	1 (1.2)	2 (1.2)	
Open field pit	5 (5.9)	6 (3.5)	
Place of waste disposal			
Burning	24 (28.2)	28 (16.5)	0.6712
Buried	32 (37.6)	30 (17.6)	
Landfill	24 (28.2)	106 (62.4)	

## 13 Determinants of Sever Acute Malnutrition

In terms of household size, children from families with five or more members had significantly higher odds of experiencing SAM compared to those from smaller families. The adjusted odds were nearly twice as high (AOR = 1.92; 95% CI: 1.24–4.08; *p* = 0.007).

Regarding birth spacing, children born less than 2 years after a sibling were found to be at substantially greater risk. These children had over four times the odds of developing SAM compared to those with a birth interval of 2 years or more (AOR: 4.11; 95% CI: 2.98–17.19; *p* = 0.003). Environmental factors were also significantly associated with the outcomes. Children living in households that relied on unprotected water sources had more than double the odds of SAM (AOR = 2.24; 95% CI: 1.37–4.11; *p* = 0.016), whereas the use of traditional cooking fuel was strongly linked to increased risk, with affected children having 6.48 times the odds of SAM compared to those using modern fuel (AOR = 6.48; 95% CI: 2.18–19.24; *p* < 0.001).

In terms of feeding practices, inappropriate timing of complementary feeding either before or after the recommended 6 months was associated with a significantly higher risk of SAM (AOR = 4.11; 95% CI: 1.87–9.15; *p* < 0.001). Regarding health status, children with comorbid illnesses had nearly twice the odds of developing SAM compared to those without (AOR = 1.96; 95% CI: 1.07–3.84; *p* = 0.048) (Table 5).

**Table 5:** Determinants of Severe Acute Malnutrition among children aged 6–23 months attending public hospitals in Mogadishu, Somalia.

Variables	Sever acute malnutrition		OR (95%CI)	AOR (95%CI)	*p* value
	Cases	Controls			
Maternal age					
5–31 years	36 (42.4)	93 (54.7)	0.64 (0.31–1.29)	0.19 (0.48–0.93)	0.702
32–49 years	49 (57.6)	77 (45.3)	1.0	1.0	
Educational level					
No formal	65 (76.5)	110 (64.7)	1.77 (0.98–3.20)	1.29 (0.59–2.83)	0.514
Formal	20 (23.5)	60 (35.3)	1.0	1.0	
Maternal occupational status					
Employed	20 (23.5)	52 (30.6)	1.0	1.0	
Unemployed	65 (76.5)	118 (69.4)	1.43 (0.78–2.60)	1.20 (0.57–2.51)	0.618
Family size					
≥ 5	67 (78.8)	91 (53.5)	3.23 (1.77–5.89)	1.92 (1.24–4.08)	0.007*
< 5	18 (21.2)	79 (46.5)	1.0	1.0	
Place of child birth					
Home	51 (60.0)	58 (34.1)	2.89 (1.69–4.96)	1.14 (0.53–2.44)	0.726
Health institution	34 (40.0)	112 (65.9)	1.0	1.0	
Consoling during PNC					
Yes	43 (50.6)	102 (60.0)	1.0		
No	42 (49.4)	68 (40.0)	0.68 (0.40–1.15)	1.41 (0.68–2.91)	0.345
Birth interval					
< 2 years	82 (96.5)	150 (88.2)	3.64 (1.05–12.63)	4.11 (2.98–17.19)	0.003*
≥ 2 years	3 (3.5)	20 (11.8)	1.0	1.0	
Sources of water					
Protected	75 (88.2)	163 (95.9)	1.0	1.0	
Unprotected	10 (11.8)	7 (4.1)	3.11 (1.13–8.47)	2.24 (1.37–4.11)	0.016*
Types of cooking fuel					
Modern	5 (5.9)	80 (47.1)	1.0	1.0	
Traditional	80 (94.1)	90 (52.9)	14.22 (5.48–36.86)	6.48 (2.18–19.24)	< 0.001*
Methods of waste disposal					
Proper	25 (29.4)	108 (63.5)	1.0	1.0	
Improper	60 (70.6)	62 (36.5)	4.18 (2.38–7.32)	1.62 (0.77–3.41)	0.203
Gender of child					
Male	36 (42.4)	93 (54.7)	1.0	1.0	
Female	49 (57.6)	77 (45.3)	1.64 (0.97–2.78)	1.07 (0.54–2.10)	0.84
History of malnutrition					
Yes	41 (48.2)	65 (38.2)	1.51 (0.89–2.55)	1.15 (0.58–2.27)	0.683
No	44 (51.8)	105 (61.8)	1.0		
Exclusive breastfeeding					
Yes	51 (60.0)	135 (79.4)	1.0	1.0	
No	34 (40.0)	35 (20.6)	2.57 (1.45–4.55)	1.64 (0.76–3.54)	0.201
Initiation of complementary feeding					
At six months	50 (58.8)	152 (89.4)	1.0	1.0	
Before or after six months	35 (41.2)	18 (10.6)	5.91 (3.07–11.34)	4.11 (1.87–9.15)	< 0.001*
Comorbidities status					
Yes	52 (61.2)	76 (44.7)	1.94 (1.14–3.31)	1.96 (1.07–3.84)	0.048*
No	33 (38.8)	94 (55.3)	1.0	1.0	

*Note:* Bold values indicate statistically significant.

## 14 Discussion

This case‐control study examined the factors contributing to SAM among children aged 6–23 months in public hospitals in the Benadir region. The study identified several determinants of SAM, including household size, short birth intervals, unprotected water sources, the use of traditional cooking fuel, improper timing of complementary feeding and the presence of comorbidities. In terms of household demographics, children from families with five or more members had nearly double the odds of developing SAM. This finding aligns with studies conducted in Karat [20] and Bahir Dar City public hospitals, Northwest Ethiopia [19]; which indicated that children from families with more than five members were more likely to experience acute malnutrition compared to those from families with fewer than five members. A study from Ahmedabad, India also found that children living in larger families were more likely to suffer from acute malnutrition than those from smaller households [21]. This may be explained by the fact that as family size increases, the portion of food available for each child often decreases, which may adversely affect the nutritional well‐being of children. Large family sizes can strain limited household resources, making it harder to provide enough nutritious food for everyone, as there's greater competition for what little is available [22]. However, this finding contrasts with a study from East Gojjam zone, Ethiopia, which reported that larger household sizes actually served as a protective factor against child malnutrition [18]. Regarding health status, children with comorbid illnesses had nearly twice the odds of developing SAM compared to those without. Although comorbidities were associated with SAM, it is important to note that these conditions may also be consequences of malnutrition rather than independent causal risk factors, indicating a bidirectional relationship.

Short birth intervals (< 2 years) were strongly associated with SAM in this study (AOR = 4.1). This aligns with findings in Ethiopia that show short intervals significantly increase the likelihood of child under‐nutrition (stunting/underweight) and that some of the effect is mediated by maternal anaemia and small birth size [23]. This association may be explained by the fact that as birth order increases, the number of dependents also rises, which can lead to inadequate feeding and competition for limited household resources. In addition, parents often devote more time and attention to newborns, which may reduce the care provided to older children. Another possible reason is limited awareness or practice of appropriate birth spacing methods among parents.

Use of unprotected water sources was also increased the risk of SAM. Several studies demonstrate that unsafe drinking water, inadequate sanitation and hygiene practices are strongly associated with child malnutrition outcome. A systematic review also noted mixed but suggestive evidence that WASH interventions may contribute to acute malnutrition prevention or recovery [24, 25]. In the context of Somalia, where only ~52% of the population access basic water supply and open defecation remains common, the link between unsafe water and child under‐nutrition is particularly plausible [26], which is also increased the risk of SAM.

This study found that introducing complementary foods either before or after 6 months of age significantly increased the risk of SAM, with a more than fourfold higher likelihood compared to timely introduction at 6 months. Similar findings have been reported in studies from India [21], and Nepal [9]. Both early and delayed initiation of complementary feeding were associated with an increased risk of SAM; however, delayed initiation showed a stronger association, likely due to prolonged nutrient deficiency beyond 6 months. Early introduction of complementary food can reduce breast milk intake and compromise the infant's immune protection, leading to a higher risk of infections such as respiratory illnesses, eye infections, and malaria. In contrast, late introduction may result in inadequate nutrient intake, as breast milk alone becomes insufficient beyond 6 months. Additionally, poor weaning and feeding practices increase the risk of diarrhea and low energy intake, contributing to malnutrition [22]. Finally, children from households that primarily used solid fuels were more likely to develop SAM compared to those from households using cleaner energy sources. This finding is consistent with a study conducted in Ethiopia [27], which reported that children living in homes that relied on biomass fuels such as firewood, charcoal, and dung had a higher risk of undernutrition due to household air pollution and its associated health effects.

## 15 Implications

The findings of this study highlight the need for integrated, multisectoral interventions to address the underlying causes of SAM in Somalia. Strengthening maternal and reproductive health services, including family planning and nutrition education, is essential to reduce the impact of large family size and short birth intervals. Expanding access to safe water, sanitation, and clean cooking fuels should be prioritized to minimize infection risks and household air pollution that contribute to malnutrition. In addition, nutrition counseling on appropriate breastfeeding and complementary feeding practices during antenatal and postnatal care is crucial for improving child health outcomes. The strong association between comorbid illnesses and SAM underscores the importance of integrating nutrition, disease prevention, and early treatment within child health programs. These findings provide practical evidence to guide Somalia's National Nutrition Strategy and strengthen community‐based interventions aimed at reducing child malnutrition and mortality.

## 16 Limitations

While this study provides valuable insights into the predictors of SAM among children in Mogadishu, several limitations should be acknowledged. First, as a hospital‐based case–control study, the findings may not fully represent the general population, which limits their generalizability to community settings. Future community‐based research could provide broader and more representative data. Second, certain information such as breastfeeding duration, complementary feeding practices, and household characteristics relied on maternal recall, which may have introduced recall or social desirability bias. Third, anthropometric assessment relied primarily on MUAC measurements, and other indicators such as weight‐for‐length z‐scores (WLZ) were not included, which may limit comprehensive nutritional assessment. Finally, maternal auxologic data such as body mass index (BMI) and height were not collected, limiting the ability to assess intergenerational nutritional effects.

## 17 Conclusion

This study identified key determinants of SAM among children aged 6–23 months attending public hospitals in Mogadishu, Somalia. The findings demonstrate that SAM is not caused by a single factor but rather by the combined effects of household, maternal, environmental, and child‐related conditions. Specifically, large family size, short birth interval, use of unprotected water sources, dependence on traditional cooking fuels, inappropriate complementary feeding practices, and the presence of comorbidities were independently associated with an increased risk of SAM. These results highlight the urgent need for integrated and multisectoral interventions to address both the immediate and underlying causes of child malnutrition. Promoting optimal birth spacing through accessible family planning services, improving maternal and child nutrition education, and strengthening exclusive and continued breastfeeding practices should be prioritized. In addition, ensuring safe water and sanitation, encouraging the adoption of clean cooking energy, and improving the management of childhood illnesses are essential steps toward breaking the malnutrition–infection cycle. At the policy level, the findings provide valuable evidence to inform Somalia's National Nutrition and Public Health Strategies. Efforts should focus on expanding community‐based nutrition programs, enhancing WASH (Water, Sanitation, and Hygiene) infrastructure, and promoting sustainable energy use to create a healthier environment for child growth. Furthermore, strengthening collaboration between health, education, and environmental sectors will ensure that interventions are comprehensive and sustainable.

## Author Contributions

**Muna Hassan Warsame:** conceptualization, methodology. **Sade Bashir Hassan:** formal analysis, data curation. **Jamal Hassan Mohamoud:** conceptualization, methodology. **Ahmed Mahad Sheikh Mohamed:** Writing – original draft. **Mohamed Hussein Adam:** formal analysis, data curation. **Bashiru Garba:** writing – review and editing. **Mohamed Adam Mahamud:** writing – original draft.

## Funding

The authors have nothing to report.

## Conflicts of Interest

The authors declare no conflicts of interest.

## Author Responsibility Statement

All authors have read and approved the final version of the manuscript. Jamal Hassan Mohamoud had full access to all data in the study and takes responsibility for the integrity of the data and the accuracy of the analysis.

## AI Disclosure

AI‐assisted tools were used for language editing and formatting of the manuscript. No AI tools were used in data analysis or interpretation.

## 1 Transparency Statement

Jamal Hassan Mohamoud affirms that this manuscript is an honest, accurate, and transparent account of the study being reported; no important aspects have been omitted.

## Data Availability

The data that support the findings of this study are available on request from the corresponding author. The data are not publicly available due to privacy or ethical restrictions.
